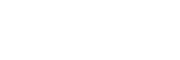# Impact of the sars‐COV2 pandemic on cognitive frailty in older adults: The role of social support

**DOI:** 10.1002/alz.089561

**Published:** 2025-01-09

**Authors:** Andrea Fabbo, Barbara Manni, Emanuele Rocco Villani

**Affiliations:** ^1^ Local Agency for Health of Modena (AUSL), Modena Italy; ^2^ UOC Geriatria ‐ Disturbi Cognitivi e Demenza; AUSL Modena, Modena Italy

## Abstract

**Background:**

Social restrictions and closures of services due to COVID‐19 pandemic had a negative impact on the social inclusion and well‐being of older people. In fact, older adults present risk factors both in terms of health ‐ such as frailty or multimorbidity ‐ and in terms of quality of life ‐ for example institutionalization ‐ and poor social support. The main objective is to evaluate whether social support had the role of an effect modifier on the incidence of cognitive frailty. Material: single‐center, cohort study, based on retrospective data collected during the emergency phase of the COVID‐19 pandemic and on a re‐evaluation performed in the post‐emergency phase. To evaluate social support, Cohen’s Social Support Scale (CSSS) was used, whose higher scores identify subjects with a low level of social support. To screen for cognitive impairment, the Mini Mental State Assessment (MMSE) was used, with an adjusted score of 26 ore less indicative of cognitive impairment. Frailty was assessed through a frailty index and cognitive frailty was defined as the co‐occurence of both cognitive impairment and physical frailty.

**Methods:**

patients belonging to the frailty clinic of the Modena AUSL with a first evaluation carried out between October 2020 and June 2021 and with data relating to social support are re‐evaluated between october and December 2023.

**Results:**

105 participants were enrolled. 68 (64.7%) were found to be frail at baseline. There was no statistically significant difference in mean CSSS between non‐frail and frail patients (8.7 ± 2.1 vs. 9.3 ± 2.2, p = 0.173). At December 2023, 50 patients were reevaluated. During follow‐up, 25 out of 37 patients developed cognitive frailty. The OR of cognitive frailty was higher when basal Cohen’s social support scale was higher (1.91, 95% CI 1.08‐3.25). Discussion: the impact of the pandemic, in terms of reduced social support should be assessed in the transition phase between the emergency and post‐emergency phase

**Conclusions:**

investments should be made to implement psychosocial support interventions to reduce further incidence of cognitive frailty among older adults